# The residual rate of HPV and the recurrence rate of CIN after LEEP with negative margins: A meta-analysis

**DOI:** 10.1371/journal.pone.0298520

**Published:** 2024-03-14

**Authors:** Yong Lin, Yan Long, Jin He, Qinqin Yi

**Affiliations:** Luzhou Maternal and Child Health Hospital (Luzhou Second People’s Hospital), Luzhou City, Sichuan Province, China; Sapienza University of Rome: Universita degli Studi di Roma La Sapienza, ITALY

## Abstract

**Background:**

HPV is detected in up to 47% of CIN and up to 70% of cervical cancers. It can cause intraepithelial neoplasia, which can eventually progress to invasive carcinoma. Almost all cervical cancers are caused by HPV. Therefore, it is especially important to treat high-risk HPV. For patients who have undergone LEEP surgery, this procedure can effectively treat CIN. However, it has not been studied in a meta-analysis whether HPV remains after the surgery and whether residual HPV increases the recurrence risk of CIN. To address this gap, our study collected all relevant literature to investigate the residual rate of HPV and its potential influence on the recurrence rate of CIN. We aim to provide valuable recommendations for clinicians and patients.

**Methods:**

The Cochrane Library, EMBASE, and PubMed databases were searched from the establishment of the database until October 2023. Stata 12.0 software was used for the statistical analysis.

**Results:**

Twelve studies were included, with a total sample size of 1192 cases. The meta-analysis found that the recurrence rate of CIN was quite low [95% CI = 0.5% (0.001, 0.012); P = 0.006] when the margins were negative after LEEP and there was no residual HPV. When HPV was present, the recurrence rate of CIN was significantly higher [95% CI = 18% (0.089, 0.291), P = 0.000], even if the margins were negative. The recurrence rate of CIN with residual HPV was 3.6 times higher than the recurrence rate of CIN without residual HPV. The residual rate of HPV after LEEP with negative margins was 22.7% [95% CI (0.167, 0.294), P = 0.000], which remained relatively high.

**Conclusion:**

This meta-analysis found that the recurrence rate of CIN without residual HPV and with negative margins after LEEP was quite low, at 0.5%. However, when HPV was residual, the recurrence rate of CIN significantly increased to 18%, even if the margins were negative. The residual rate of HPV was 22.7%, even when the margins were negative after LEEP.

## 1. Background

Human papillomavirus (HPV) is a non-enveloped DNA virus that belongs to the genus Papillomavirus, specifically to the genus Papillomavirus A of the family Papillomaviridae. HPV is known to cause the proliferation of squamous epithelial cells. HPV is detected in up to 47% of cervical intraepithelial neoplasia (CIN) [1] and up to 70% of cervical cancers [2]. It can cause intraepithelial neoplasia, which can eventually progress to invasive carcinoma. Almost all cervical cancers are caused by HPV, which is the primary factor responsible for approximately 5.2% of human cancers worldwide. Cervical cancer is ranked as the fourth most common cancer in women globally [3–5]. Although prophylactic vaccines have been successful in preventing HPV infection in healthy individuals, they do not cure or eliminate HPV infections or cervical intraepithelial neoplasia (CIN).

Women with persistent high-risk HPV infection are susceptible to developing CIN, which can progress to cervical cancer if not detected and treated [6, 7]. The cervical loop electrosurgical excision procedure (LEEP) is a method used to treat CIN and can also eliminate HPV [8].

However, no meta-analysis has shown the residual rate of HPV or the recurrence rate of CIN after LEEP with negative margins. Additionally, it is unclear whether the presence of residual HPV increases the recurrence risk of CIN. It is crucial for patients to understand the residual rate of HPV after LEEP and the recurrence rate of CIN.

The aim of this meta-analysis was to investigate the residual rate of HPV and the recurrence rate of CIN in patients with negative margins after LEEP. Additionally, the study aimed also to determine whether the presence of residual HPV increases the recurrence risk of CIN.

## 2. Method

### 2.1 Inclusion and exclusion criteria

Inclusion criteria included: 1. Randomized controlled trials (RCTs), observational studies, comparative studies, clinical trials, multi-center studies, and controlled studies. 2. The intervention was limited to LEEP. 3. The outcomes included the residual rates of HPV and the recurrence rates of CIN after LEEP. Exclusion criteria were as follows: 1. Animal experiments, cellular studies, reviews, meta-analyses, case reports, and letters. 2. Literature that repeatedly uses the same data. 3. Literature with incorrectly usable data. 4. The patients had received HPV vaccination.

### 2.2 Literature search strategy

The Cochrane Library, EMBASE, and PubMed databases were searched from the establishment of the database until October 2023. Additionally, a comprehensive review of the references cited in the included articles was conducted. We conducted a manual literature search using the search terms "LEEP" and "HPV". The language was limited to English.

### 2.3 Data extraction and assessment of literature quality

According to the search strategy, two evaluators (Long Yan and Yong Lin) independently reviewed all the literature to identify eligible studies. Abstracts of all the literature were reviewed to identify eligible studies. Full reports of all eligible studies were obtained to assess whether they met the predefined inclusion criteria. Disagreements were documented, and consensus was reached among all the authors. For the eligible studies, we collected relevant information such as authors, year of publication, age, sample size, duration of follow-up, residual rate of HPV, and recurrence rate of CIN after LEEP. The quality of the literature was assessed by two evaluators independently applying the Methodological Index for Non-Randomized Studies (MINORS). In cases where data was missing or more details were needed, we attempted to retrieve the missing data by contacting the first author as necessary.

MINORS is a quality assessment tool that can be used to evaluate observational studies, non-randomized controlled trials (non-RCT) studies, and other types of research. In this study, only the results of the single-arm ratio were assessed; therefore, only the first eight indicators were used. These indicators include the following: whether the study’s purpose is clear, the consistency of the included patients, whether the expected data has been collected, whether the endpoint indicators appropriately reflect the study’s purpose, the objectivity of the evaluation of the endpoint indicators, the adequacy of follow-up, whether the study dropout rate is less than 5%, and whether the sample size has been estimated. Each item is scored on a scale of 0–2. A score of 0 indicates unreported, a score of 1 indicates reported but inadequate, and a score of 2 indicates reported and adequate. The ideal score is 16 [9].

### 2.4 Statistical analysis

Stata 12.0 software was used for the statistical analysis. When I^2 was greater than 50% or P was greater than 0.1, it indicated that there was heterogeneity among the studies. Subgroup analysis was conducted to investigate the factors contributing to homogeneity from both clinical and methodological perspectives. After excluding the influence of obvious clinical heterogeneity, a random-effects model was used for the analysis. If there was significant heterogeneity between the two groups or clinical heterogeneity, descriptive analysis was used. When I^2 was less than 50% or P was less than 0.1, it was considered that there was no heterogeneity among the studies, and the studies were combined using the fixed-effects model. Sensitivity analysis and publication bias analysis were also conducted. Because the incidence of complications in this meta-analysis was relatively small, the data was transformed using the double inverse chord transformation method and then statistically combined using the "metaprop" method.

### 2.5 Sensitivity analysis

Sensitivity analyses of the included studies were conducted using the "metainf" command in Stata 12.0 software.

### 2.6 Publication bias

Funnel plots were created using the "metafunnel" command in Stata 12.0 software. Publication bias was assessed subjectively by examining the symmetry of the funnel plots. If the funnel plot is symmetrical, it suggests that there is no publication bias. The Egger test is commonly used to objectively assess publication bias, with a p-value greater than 0.05 indicating no publication bias.

### 2.7 Ethical considerations

All analyses were based on published studies and did not require ethical approval or patient consent.

## 3 Results

### 3.1 Literature screening process and basic characteristics of included literature

**Fig 1:**
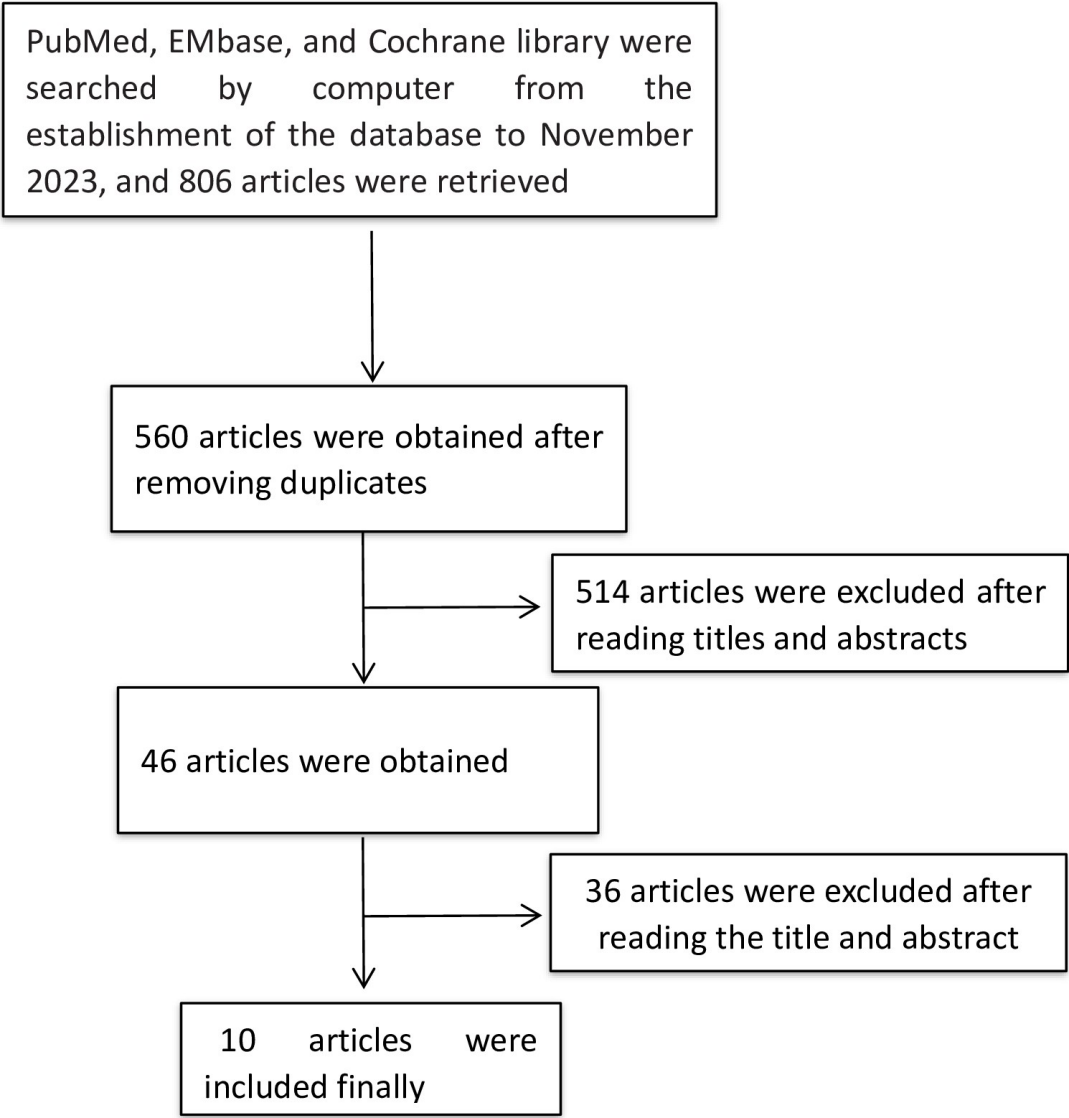
The process of literature screening.

A total of 806 relevant literatures were obtained during the initial screening. After reviewing the titles, abstracts, and full texts, 10 studies were ultimately included [10–19], which involved a total of 1292 patients. The process of literature screening process is shown in Fig 1, and the essential details of the included studies are shown in Table 1.

**Table 1:** The essential details of the included studies.

study	Age	study type	sample size	Interventions	Outcome*	Time (months)
Lei Zhang 2016	38.2±8.56	Single-arm	100	LEEP	1、2	3 months after surgery
Nasuh Utku Dogan 2011	39.36±7.97	clinical trials	37	LEEP	1	6 months after surgery
Young-Tak Kim 2010	40.7±8.8	Single-arm	287	LEEP	1、2、3	6 months after surgery
B. PRATO 2008	-	Single-arm	115	LEEP	1、2	6-12months after surgery
Laurențiu Pirtea 2016	-	Single-arm	85	LEEP	1	6 months after surgery
R. Du 2013	41.14±8.81	Single-arm	141	LEEP	1、2、3	6 months after surgery
Maria Teresa Bruno 2019	39.3±8.7	Single-arm	162	LEEP	1、2	6 months after surgery
Aeli Ry 2012	39.3±8.7	Single-arm	134	LEEP	1、2	3 months after surgery
Kyehyun Nam et al 2009	39.9	Single-arm	263	LEEP	1	6 months after surgery
V. Houfflin 2003	34.7±9.17	Single-arm	131	LEEP	1、3	18 months after surgery

*1:The residual rate of HPV after LEEP with negative margins, 2:The recurrence rate of CIN of with residual HPV AND negative margins after LEEP, 3:The recurrence rate of CIN without residual HPV and with negative margins after LEEP.

### 3.2 Quality assessment

The 10 single-arm studies had MINORS scores ranging from 11 to 15. All studies used the standardized tool MINORS, and the quality scores of the articles included in the final analysis ranged from 11 to 15, as assessed by the MINORS quality assessment checklist. Eight studies were classified as high-quality (scores of 12 and above), and the remaining studies were considered fair-quality (scores of 11). The MINORS scores of the studies are shown in Table 2.

**Table 2:** MINORS scores of the studies.

study	I	II	III	IV	V	VI	VII	VIII	total
Lei Zhang 2016	2	2	1	1	2	1	1	1	11
Nasuh Utku Dogan2011	2	2	2	1	2	1	0	1	11
Young-Tak Kim2010	2	2	2	1	2	2	2	2	15
B. PRATO 2008	2	2	2	1	2	2	2	1	14
Laurențiu Pirtea 2016	2	2	2	1	2	2	2	1	14
R. Du 2013	2	2	1	1	2	2	2	0	12
Maria Teresa Bruno 2019	2	2	2	2	2	2	2	1	15
Aeli Ry 2012	2	2	2	1	2	2	0	1	12
Kyehyun Nam et al 2009	2	2	2	1	2	2	0	1	12
V. Houfflin 2003	2	2	2	1	2	2	0	1	12

Note:I whether the study’s purpose is clear, II the consistency of patients included, III whether the expected data has been collected, IV whether the endpoint indicators appropriately reflect the purpose of the study, V the objectivity of the evaluation of the endpoint indicators, VI the adequacy of follow-up, VII whether the study dropout rate is less than 5%, VIIIand whether the sample size has been estimated.

### 3.3 Results of the meta-analysis

#### 3.3.1 Sensitivity analysis, publication bias analysis

Sensitivity analysis: After excluding the literature one by one, all the results obtained were not affected. This indicates the robustness of the results, as shown in S1–S3 Figs.

Publication Bias: The obtained funnel plots are all symmetrical, suggesting that there is no significant publication bias in this study. Further analysis using the Egger test indicates that the possibility of publication bias in this study is low. The results are shown in S4–S6 Figs.

As the single-arm meta-analysis itself has significant heterogeneity, but sensitivity analysis and publication bias assessment revealed no literature with substantial interference in the results. Therefore, subgroup analyses were not conducted, and the random effects model was employed for the meta-analysis.

#### 3.3.2 The residual rate of HPV after LEEP with negative margins

**Fig 2:**
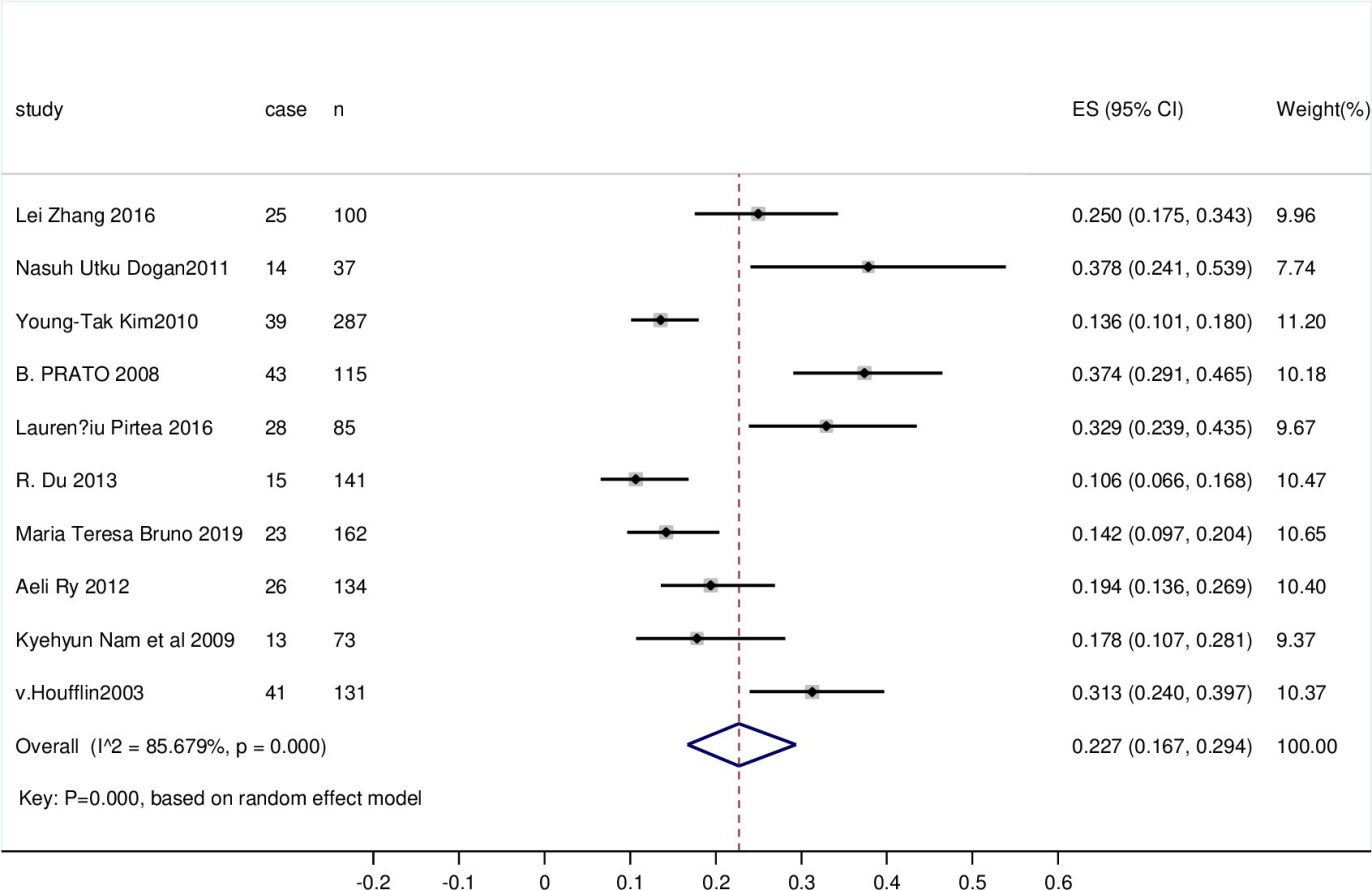
Forest plot of the residual rate of HPV after LEEP with negative margins.

All literature was included, with a total sample size of 1192 cases. Heterogeneity among studies was high (I^2 = 85.679%, P = 0.000); therefore, the random effects model was applied to combine the statistics. Egger’s test indicated no publication bias, and the results were considered to be reliable. The residual rate of HPV was 22.7% (95% CI 0.167, 0.294, P = 0.000), which remained relatively high. The observed difference was statistically significant. The forest plot is shown in Fig 2.

#### 3.3.3 The recurrence rate of CIN with residual HPV and negative margins after LEEP

**Fig 3:**
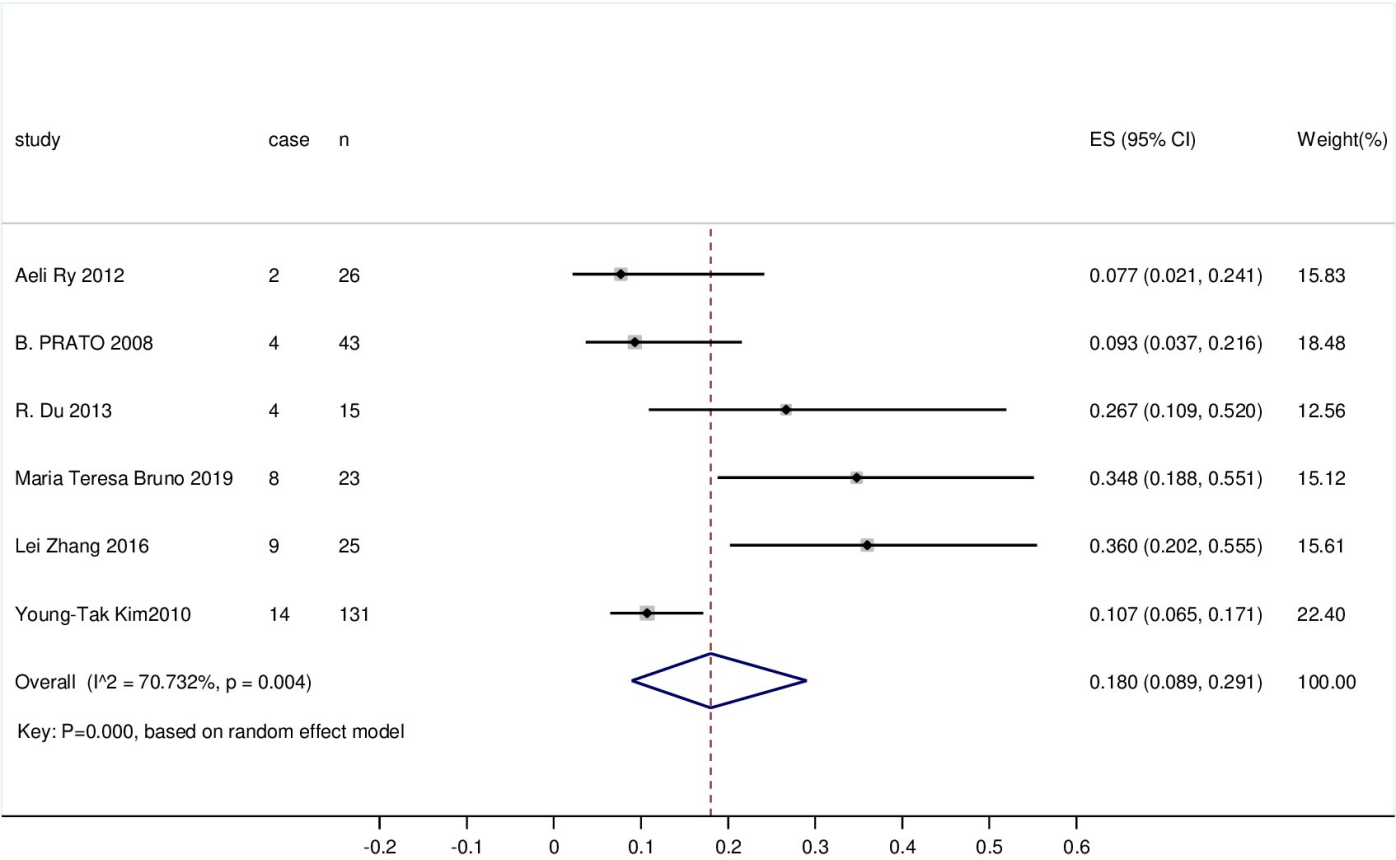
Forest plot of the recurrence rate of CIN with residual HPV and negative margins after LEEP.

263 cases were included in the literature. The study had a high level of heterogeneity (I^2 = 70.732%, P = 0.004); therefore, the random effects model was applied to combine the statistics. The recurrence rate of CIN with residual HPV and negative margins after LEEP was 18% (95% CI 0.089, 0.291, P = 0.000). The recurrence rate of CIN remained high, and the observed difference was statistically significant. The forest plot is shown in Fig 3.

#### 3.3.4 The recurrence rate of CIN without residual HPV and with negative margins after LEEP

**Fig 4:**
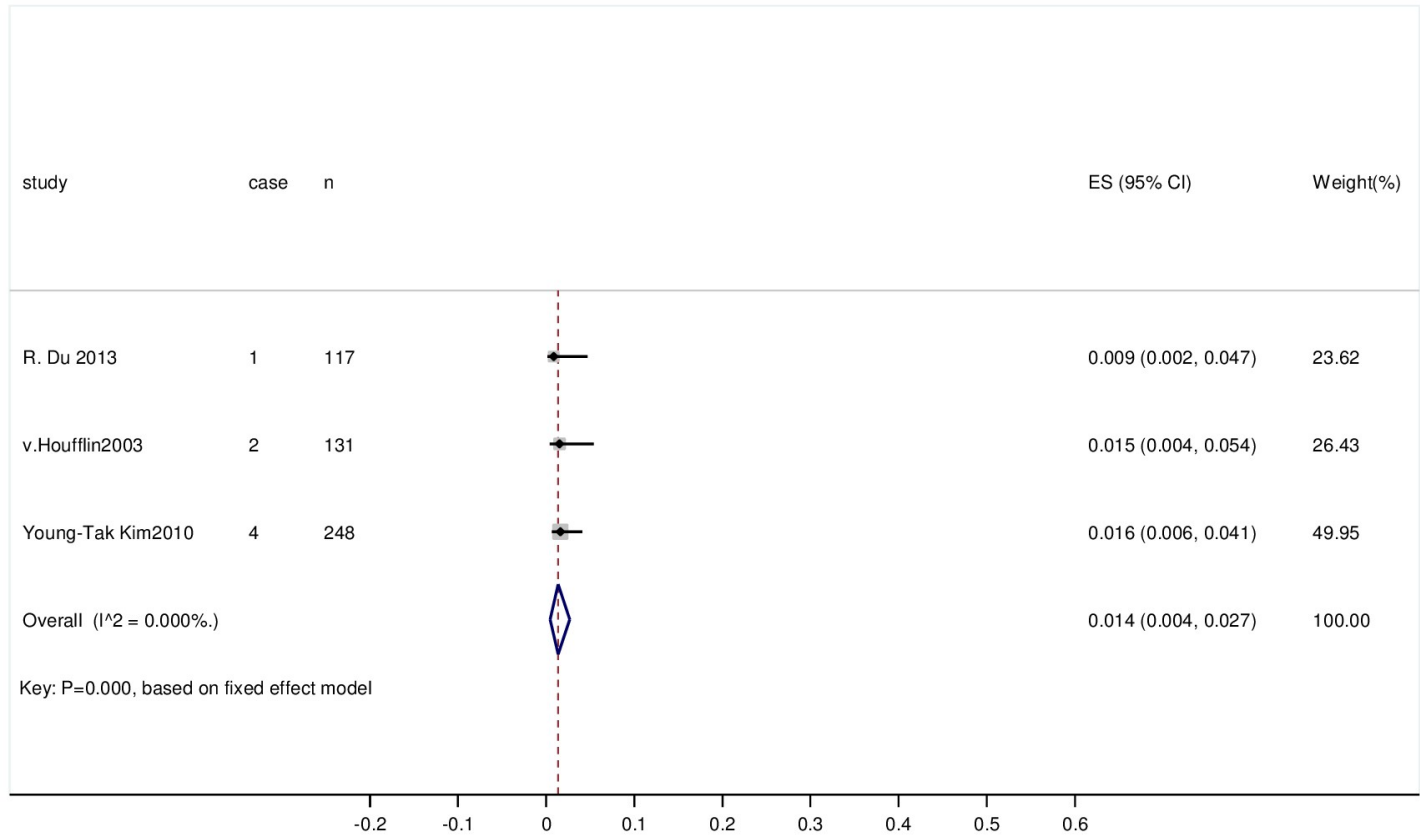
Forest plot of the recurrence rate of CIN without residual HPV and with negative margins after LEEP.

868 cases were included in the literature. The heterogeneity of the study was low (I^2 = 0%, P = 0.459); therefore, a fixed-effects model was applied to combine the statistics. The recurrence rate of CIN without residual HPV and with negative margins after LEEP was 0.5% (95% CI (0.001, 0.012); P = 0.006). The rate of CIN recurrence was low, and the observed difference was statistically significant. The forest plot is shown in Fig 4.

## 4. Discussion

Kulkarni A et al. demonstrated a significant association between human papillomavirus and positive margins with recurrence [20]. Chen LM et al. also found a higher recurrence rate in the group with positive endocervical margins compared to the group with negative margins. They identified positive endocervical margins and HR-HPV after LEEP as independent factors [21]. However, no meta-analysis has shown the residual rate of HPV and the recurrence rate of CIN after LEEP with negative margins, or whether the presence of residual HPV increases the recurrence rate of CIN.

The meta-analysis found that the recurrence rate of CIN significantly quite low when the margins were negative after LEEP and when there was no residual HPV. This finding is consistent with a previous study [22].

In contrast, the recurrence rate of CIN was significantly higher when HPV was present, even if the margins were negative. The recurrence rate of CIN with residual HPV was 3.6 times higher than the recurrence rate of CIN without residual HPV. Residual HPV is a high-risk factor for the recurrence of CIN after LEEP. A study has also shown that the persistence of HPV is one of the most significant factors in predicting the risk of recurrent CIN2+. Within one year of persistent HPV infection, the risk of CIN2+ recurrence increases with duration [23].

Various treatments for HPV have been reported, and their effectiveness has been confirmed and recognized. A study has shown that consistent condom use after LEEP significantly reduces HPV-positive rates and also appears to significantly reduce rates of CIN recurrence and biomarkers of HPV expression. Additional HPV vaccination administered at the time of treatment could further enhance the positive effects of consistent condom use [24, 25].

Another study on HPV indicates that HPV vaccination in HPV-positive patients may increase the chances of HPV remission and could be a valuable treatment approach for such patients [26].

It is necessary to reexamine for HPV after LEEP. As widely known, Pap tests are notoriously insensitive, detecting only about 40% of cases. To address the sensitivity gap, the human papillomavirus DNA molecular test was incorporated into the Pap test. This combination achieved a sensitivity of approximately 90%. Several studies have focused on protein biomarkers. These tests can identify the potential progression from preinvasive to invasive lesions. In particular, low molecular weight proteins and proliferation markers (Ki-67) that bind to cyclin-dependent kinases 4 and 6 (p16ink4a) appear to identify indeterminate diagnoses of atypical squamous cells of undetermined significance (ASC-US) or low-grade squamous intraepithelial lesions (L-SIL) [27].

If any residual HPV is detected, timely treatment should be administered. The goal of global cervical screening programs, whether based on cytology or HPV (human papillomavirus) biomarkers, is to focus on the early detection and treatment of preinvasive lesions, ultimately reducing cervical cancer morbidity and mortality [28–30].

Even if HPV is not found after LEEP, it is very important to prevent the development of precancerous lesions. The prevention and early detection of precancerous lesions are essential goals for improving patient outcomes and reducing the long-term costs of managing gynecological cancers. Make screening programmes available to all women and take all necessary steps to facilitate and support the implementation of vaccination, ensuring that the program is universally accepted. And they should be applied appropriately and systematically in all countries [31]. This is our expectation and goal for the future.

## 5. Strengths and limitations

To date, this is the first time that the residual rate of HPV and the recurrence rate of CIN after LEEP with negative margins have been summarized. The analysis of evidence-based medicine in the included literature provides a higher-quality foundation for clinical practice. These results are intended to draw the attention of clinicians and provide positive recommendations for surgery and postoperative prophylaxis.

However, we still need a large sample to confirm our findings. Furthermore, there is a need for reliable biomarkers to detect the recurrence of HPV, and greater research investment is required to develop effective treatment strategies for HPV.

Such meta-analyses have certain limitations: (I) The meta-analysis of single-arm rates has its own methodological limitations, as it compares ratios rather than simple averages and lacks the design of a controlled study, which reduces the accuracy of the results. (II) Deficiencies in the quality of the published studies, such as inaccuracies in reporting the timing of pre-treatment HPV testing and the type of HPV, can result in potential publication bias and selection bias. (III) Some studies were only descriptive analyses and were unable to extract accurate clinical data. Due to the limited quantity and quality of the included studies, further research is still necessary to confirm the above conclusions.

## 6. Conclusion

This meta-analysis found that the recurrence rate of CIN without residual HPV and with negative margins after LEEP was quite low, at 0.5%. However, when HPV was residual, the recurrence rate of CIN significantly increased to 18%, even if the margins were negative. The residual rate of HPV was 22.7%, even when the margins were negative after LEEP.

## Supporting information

S1 Checklist PRISMA 2020 checklist. (DOCX)

S1 Fig The residual rate of HPV after LEEP with negative margins. (TIF)

S2 Fig The recurrence rate of CIN with residual HPV and negative margins after LEEP. (TIF)

S3 Fig The recurrence rate of CIN without residual HPV and with negative margins after LEEP. (TIF)

S4 Fig The residual rate of HPV after LEEP with negative margins. (TIF)

S5 Fig The recurrence rate of CIN with residual HPV and negative margins after LEEP. (TIF)

S6 Fig The recurrence rate of CIN without residual HPV and with negative margins after LEEP. (TIF)

## Abbreviations

HPV: human papillomavirus
CIN: cervical intraepithelial neoplasia
LEEP: loop electrosurgical excision procedure
RCTS: randomized controlled trials
MINORS: Methodological Index for Non-Randomized Studies
