# Supplementary figures and images for: The residual rate of HPV and the recurrence rate of CIN after LEEP with negative margins: A meta-analysis
Source: PLoS One. 2024 Mar 14;19(3):e0298520. doi: 10.1371/journal.pone.0298520 (PMC10939204; doi:10.1371/journal.pone.0298520)

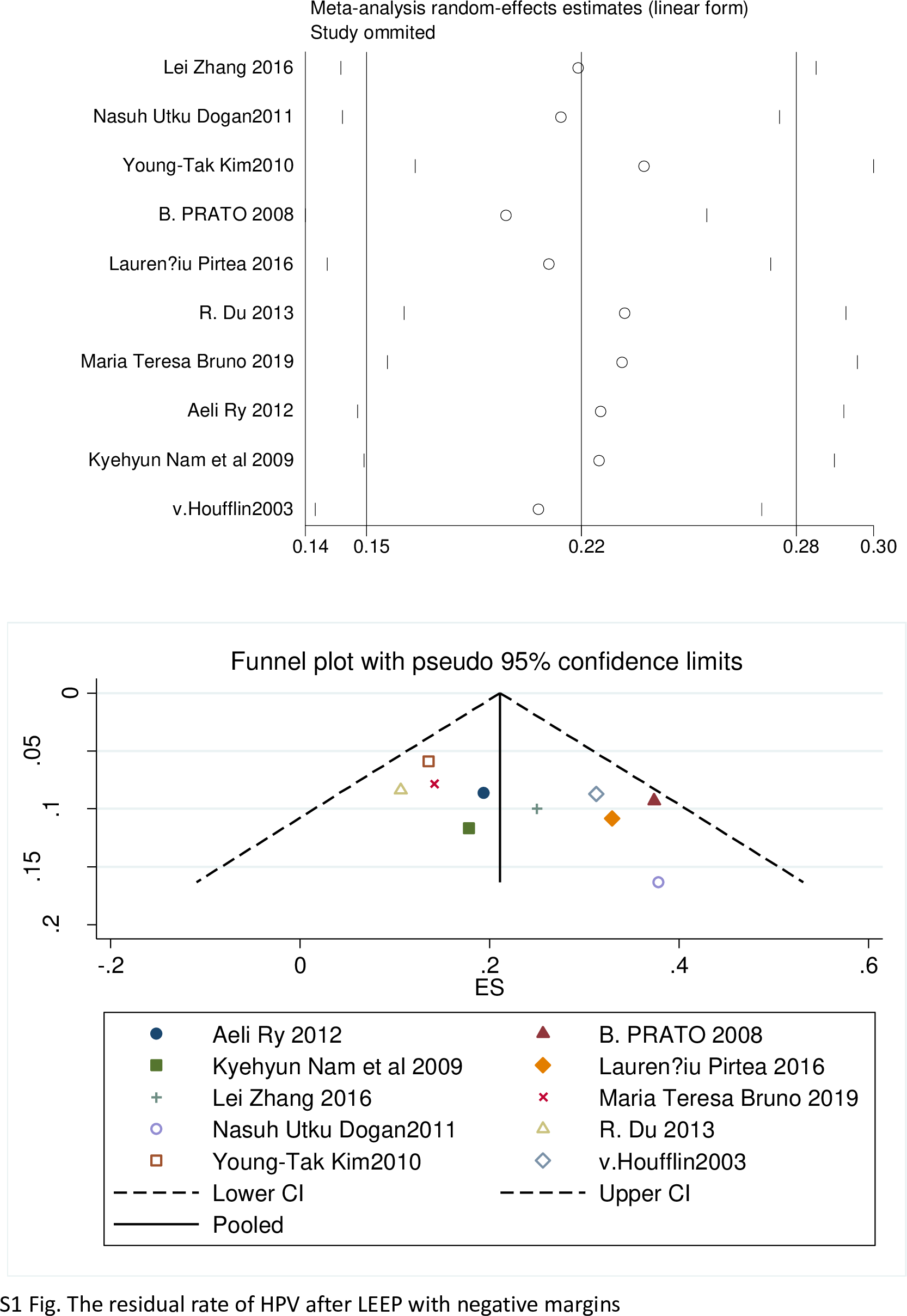

Supplement: S1 Fig — (TIF) [file pone.0298520.s002.tif]

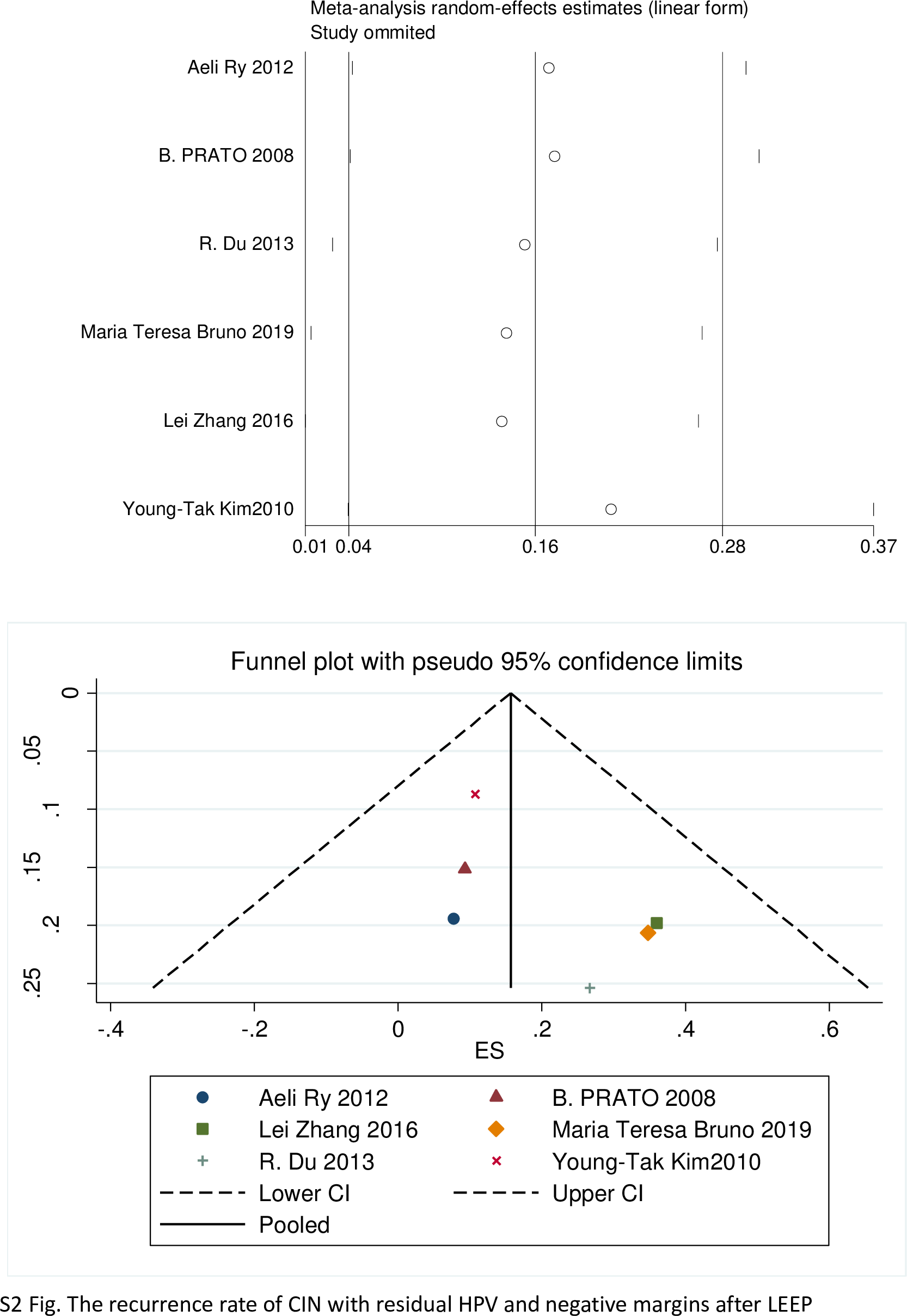

Supplement: S2 Fig — (TIF) [file pone.0298520.s003.tif]

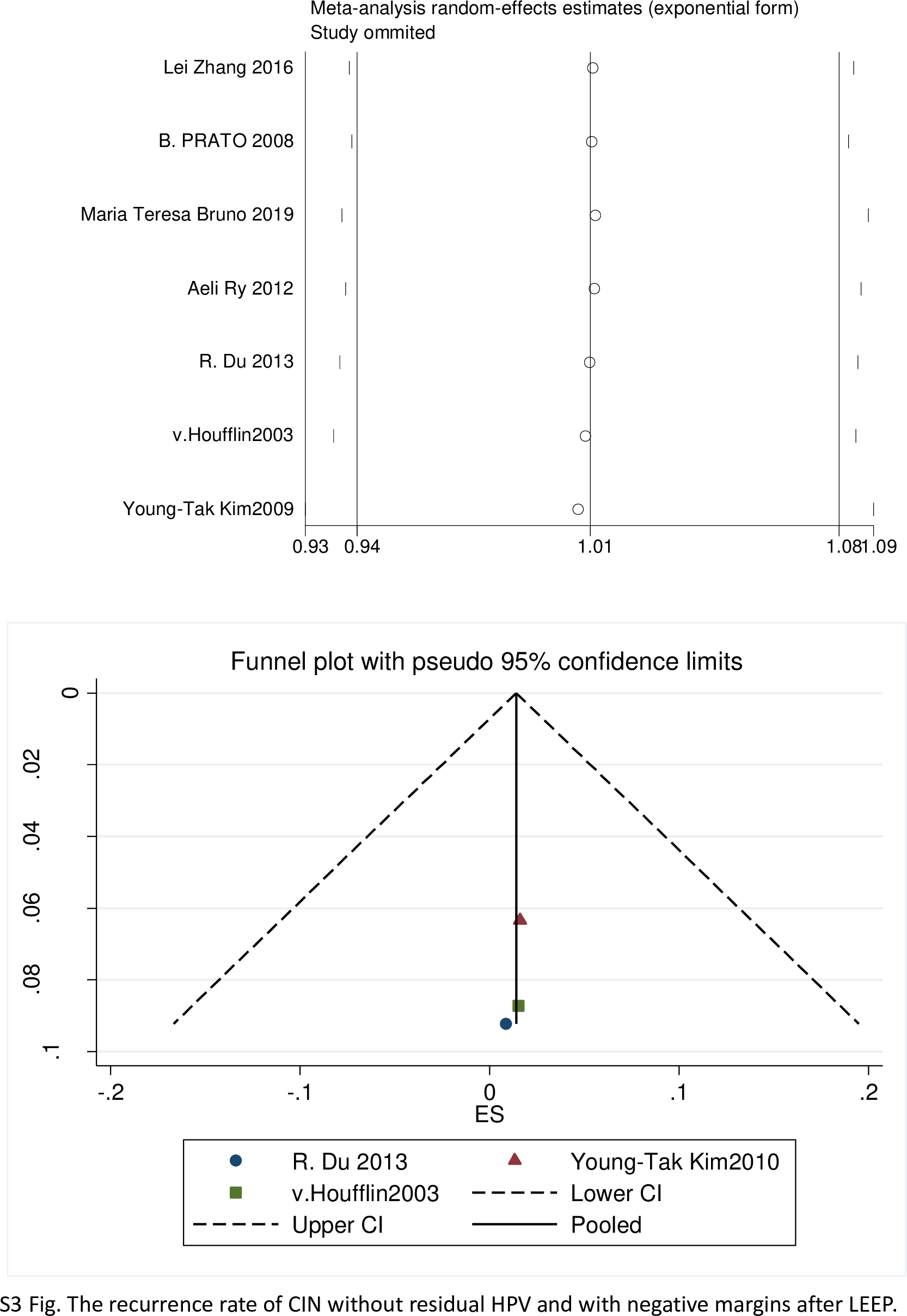

Supplement: S3 Fig — (TIF) [file pone.0298520.s004.tif]

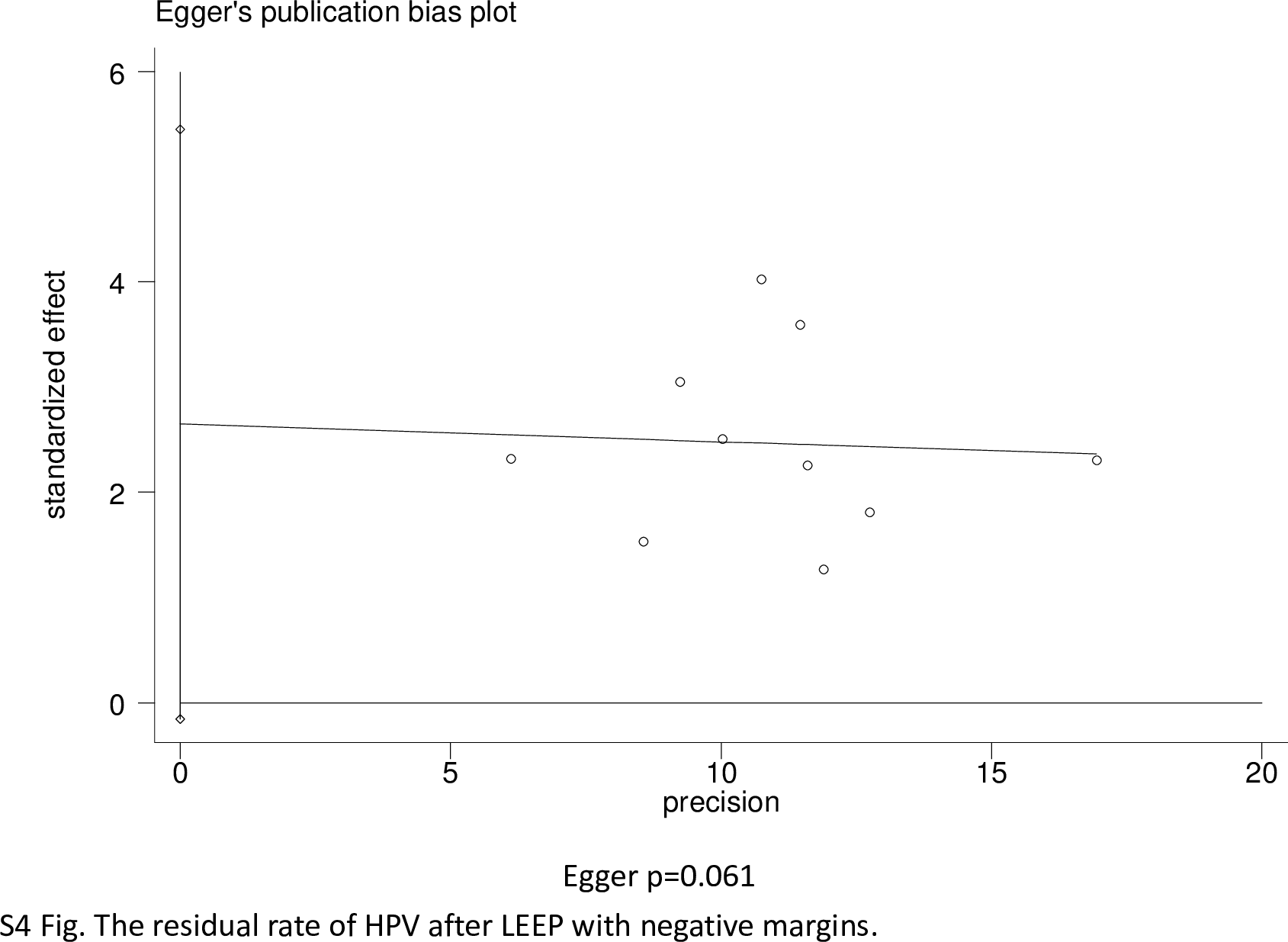

Supplement: S4 Fig — (TIF) [file pone.0298520.s005.tif]

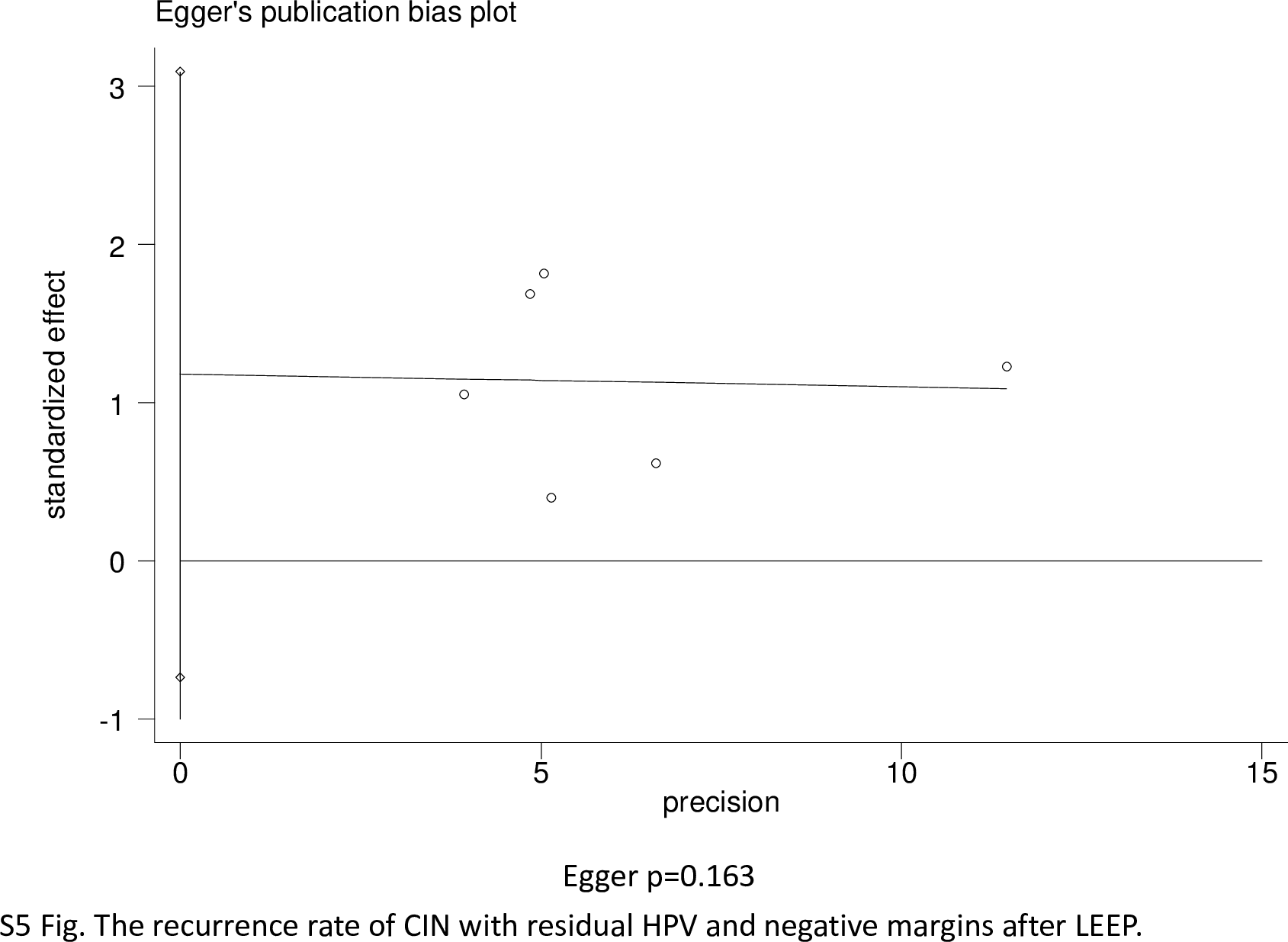

Supplement: S5 Fig — (TIF) [file pone.0298520.s006.tif]

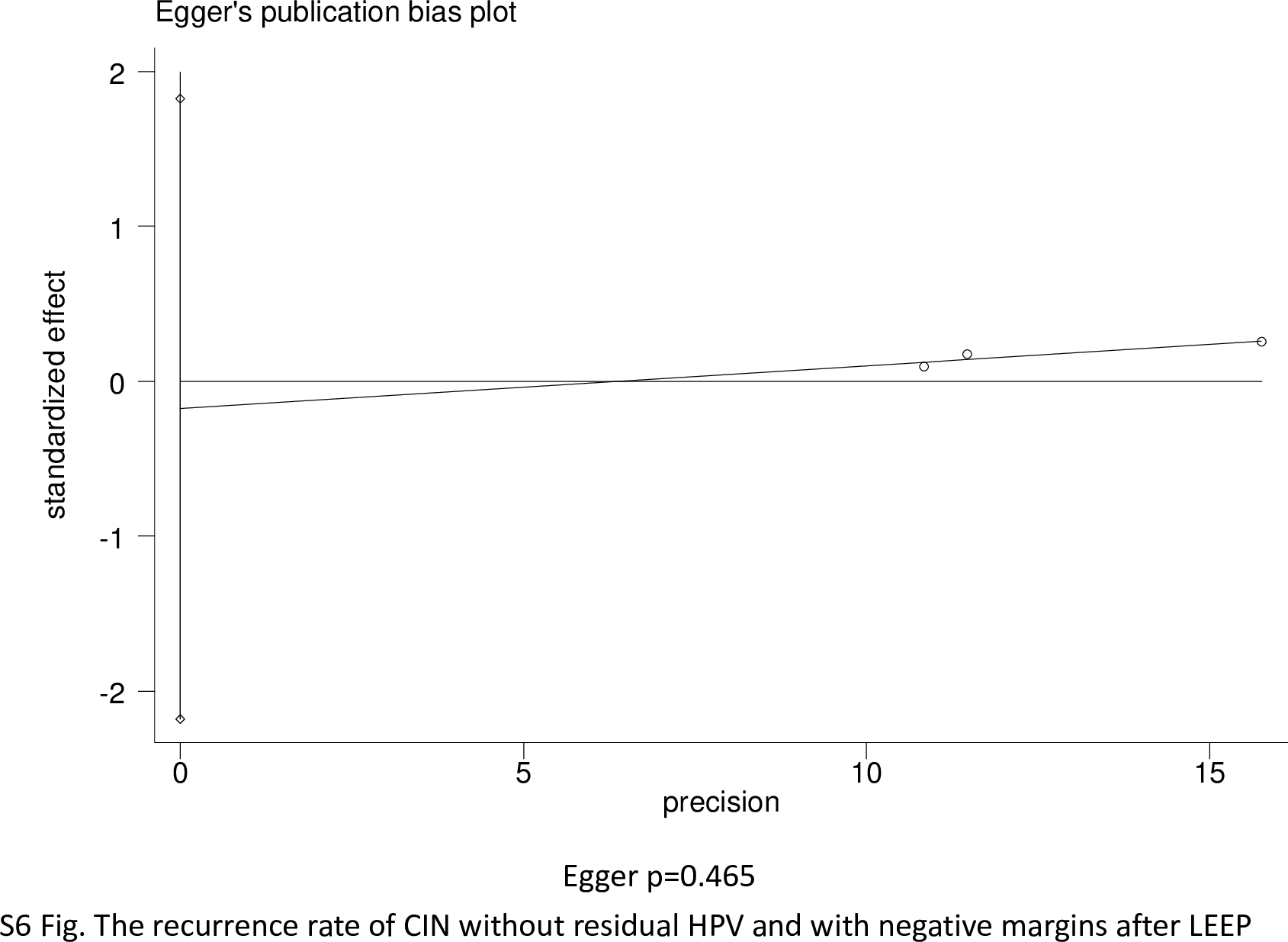

Supplement: S6 Fig — (TIF) [file pone.0298520.s007.tif]
